# Efficacy of daprodustat for patients on dialysis with anemia: systematic review and network meta-analysis

**DOI:** 10.11604/pamj.2024.47.114.37278

**Published:** 2024-03-08

**Authors:** Hammad Ali Fadlalmola, Khaled Mohammed Al-Sayaghi, Abdulqader Abdlah Al-Hebshi, Muhanad Alhujaily, Arwa Omar Alyamani, Alaa Abdulrhman Alem, Mona Hamza Syrafi, Sarah Alem, Afrah Hassan Farhat, Fathi Abdelrazig Mohamed, Hager Hassan Abdalrahman, Mohammed Abdelkrim Abdelmalik, Neimat Mahmoud Abdalrhman, Alamin Mustafa Eltayeb

**Affiliations:** 1 Department of Community Health Nursing, College of Nursing, Taibah University, Al-Madinah Al-Munawarah, Kingdom of Saudi Arabia,; 2 Department of Medical Surgical Nursing, College of Nursing, Taibah University, Al-Madinah Al-Munawarah, Kingdom of Saudi Arabia,; 3 Nursing Division, Faculty of Medicine and Health Sciences, Sana'a University, Sana'a, Yemen,; 4 Pediatric Hematology Oncology Service, Department of Pediatrics, Prince Mohammed Bin AbdulAziz Hospital, Ministry of National Guard Health Affairs, Medina, Saudi Arabia,; 5 Department of Clinical Laboratory Sciences, College of Applied Medical Sciences, University of Bisha, Bisha, Saudi Arabia,; 6 Service of Pediatric Hematology Oncology, Department of Pediatrics, King Saud Bin Abdul Azizi Collage for Health Science, Riyadh, Saudi Arabia,; 7 Princess Noorah Oncology Center, Ministry of National Guard Health Affairs, Jeddah, Saudi Arabia,; 8 Nephrology Service, Department of Medicine, College of Medicine, Taibah University, Medina, Saudi Arabia,; 9 Service of Pediatric Nephrology, Department of Pediatrics, King Salman Bin Abdulaziz Medical City, Medina, Saudi Arabia,; 10 Nahdi Medical Company, Medina, Saudi Arabia,; 11 Department of Pediatrics, King Khalid University Hospital, Riyadh, Saudi Arabia,; 12 Department of Pediatrics, Prince Mohammed Bin AbdulAziz Hospital, Ministry of National Guard Health Affairs, Medina, Saudi Arabia,; 13 College of Applied Medical Sciences, University of Hafr Albatin, Hafr Albatin, Saudi Arabia,; 14 Department of Nursing, College of Applied Medical Sciences, Shaqra University, Shaqra, Saudi Arabia,; 15 Faculty of Medicine and Health Sciences, Nursing, University of El Imam El Mahdi Kosti, White Nile, Sudan,; 16 Medical Surgical Nursing Department, Al Baha University, Baha, Saudi Arabia,; 17 Al-Neelain University, Faculty of Medicine, Khartoum, Sudan

**Keywords:** Chronic kidney, daprodustat, network meta-analysis, systematic review

## Abstract

Chronic kidney disease (CKD) is commonly complicated by anemia. Treating dialysis-dependent patients with anemia, including daprodustat and other inhibitors of prolyl hydroxylase of hypoxia-inducible factor, recombinant human erythropoietin (rhEPO), and iron supplements. We conducted this study to test our postulation; daprodustat is superior to rhEPO and other conventional treatments respecting efficacy and safety parameters. We made systematic search through PubMed, Web of Science, Scopus, and Cochrane. Seven unique trials were eventually included for systematic review; six of them with a sample size of 759 patients entered our network meta-analysis (NMA). Daprodustat 25-30 mg was associated with the greatest change in serum hemoglobin (MD=1.86, 95%CI= [1.20; 2.52]), ferritin (MD= -180.84, 95%CI= [-264.47; -97.20]), and total iron binding capacity (TIBC) (MD=11.03, 95%CI= [3.15; 18.92]) from baseline values. Dialysis-dependent patients with anemia had a significant increment in serum Hemoglobin and TIBC and a reduction in serum ferritin, in a dose-dependent manner, when administered daprodustat.

## Introduction

Chronic kidney disease (CKD) is a significant health problem that was globally estimated by about 15% of the whole population [1]. Anemia commonly complicated patients with chronic kidney disease; was reportedly estimated by about 40% in a population of 209311 individuals with CKD [2]. The leading causes of anemia were uremia and chronic inflammation that suppressed bone marrow synthesis of red blood corpuscles (RBCs), reduced RBCs lifespans, and increased serum hepcidin that sequesters iron in ferritin, a non-functional iron store [3-5]. Yet, the most crucial cause of CKD-induced anemia is decreased serum erythropoietin (EPO) [6], a hormone that was secreted normally by renal interstitial fibroblast [7-9]. Hence the conventional lines of treatments were EPO, EPO stimulating agents (ESA)s, and iron [10,11]. However, conventional treatments yielded increment of serum EPO to supraphysiologic level, which was associated with severe adverse events including venous thrombosis, stroke, cardiovascular morbidities, and ultimately death [12-15]. Additionally, the compliance of patients on dialysis with anemia was negatively affected by the parenteral and subcutaneous routes of administration of iron supplements, EPO, and ESAs [16].

Daprodustat is an orally administered small molecule that actively suppresses the prolyl hydroxylase enzyme that acts on Hypoxia-inducible factor (HIF-PH). Inhibition of HIF-PH accumulates HIF-α that forms a dimer with HIF-β; the dimer eventually enters the nucleus and drives activation of genes and transcription factors of erythropoiesis [17,18]. This state mimics hypoxia, elevates serum EPO [19-21], modulates serum hepcidin, and initiates the synthesis of proteins that uptake, transport, and store iron [19,20].

A meta-analysis by Zhong *et al*. reported that the only significant effect of daprodustat on dialysis-dependent patients was rising serum total iron-binding capacity (TIBC), with no significant effect regarding serum hemoglobin hepcidin and ferritin [21]. Another subsequent meta-analysis by Zheng *et al*. reported controversial results. In dialysis-dependent patients, daprodustat had a significant effect on serum HB and ferritin but not on serum TIBC and transthyretin saturation compared to placebo. However, compared to recombinant human EPO (rhEPO), daprodustat was reportedly associated with a significant difference respecting serum TIBC and ferritin only [22]. Moreover, no previous meta-analysis accounted for different doses of daprodustat while pooling the effects of estimates.

We aimed to conduct a network meta-analysis (NMA) to investigate the direct and indirect effects of different doses of daprodustat, rhEPO, and placebo in dialysis-dependent patients. Our primary outcome was the change in serum hemoglobin from the baseline. Our secondary outcomes were changes in serum ferritin, TIBC, and iron.

## Methods

We executed this systematic review and NMA following the recommendations of the Cochrane handbook [23]; reported it respecting the latest preferred reporting items addressed in the PRISMA checklist [24,25].

**Search strategy and data collection:** a comprehensive search was accomplished in Cochrane Library, PubMed, Web of Science, and Scopus; from the beginning to late January 2022. The subsequent search term was used in various databases: (anemia OR erythrocytopenia) and (dialysis or hemodialysis) and (daprodustat or duvroq or “GSK-1278863” or “GSK1278863”); was modified regarding requirements of each database. We gathered retrieved records in version nine of EndNote; exported the results in an Excel sheet to be screened.

**Selection criteria:** the identified studies were eligible if they matched these stipulated criteria: (1) population: patients on dialysis with anemia who were set to dialysis; (2) intervention: different doses of daprodustat; (3) comparable: placebo or any other treatment used for anemia of CKD; outcomes: serum HB (primarily), serum ferritin, TIBC, and serum iron (secondarily). We only included randomized clinical trials (RCTs) that reported human data in English.

**Data extraction:** two separate authors extracted the subsequent data in an Excel sheet: 1) summary of the eligible trials: study ID (last name of first author/publication date), site, design, study arms, sample size, doses, route of administration, inclusion criteria, exclusion criteria, duration of follow-up, primary endpoints, and conclusions; 2) characters of the enrolled patients at the basis: study arms, number of patients in each arm, mean age, percentage of females, weight, body mass index, serum iron, HB, TIBC, ferritin, transthyretin saturation (TSAT), hepcidin, race, history of prior diseases, and type of dialysis; 3) outcomes: serum HB, ferritin, TIBC, and serum iron; 4) domains of the quality assessment.

**Quality assessment:** the quality of eligible trials was assessed by the Cochrane risk of bias tool, version one, which was consisted of these domains: selection bias (sequence generated randomly and concealed allocation), blinded assessors (detection bias), blinded personnel, and participants (performance bias), selective or incomplete reporting, and other bias [26]. Two authors judged every domain independently; discrepancies were resolved by a third one.

**Statistical analysis:** we pooled the data in this frequentist NMA using the netmeta package of RStudio. We exhibited the continuous data as mean difference (MD) and 95% confidence interval (CI). Any substantial heterogeneity was appraised using the Chi-squared (Q2) test; and was quantified by the I-squared (I2) test. We considered heterogeneity statistically significant if the P-value of Q2 was less than 0.1 or the I2 test valued over 50%. Heterogenous studies were treated by a random effect model. The arms of analyzed interventions were ordered from the highest to lowest effect in a league table.

## Results

**Figure 1:**
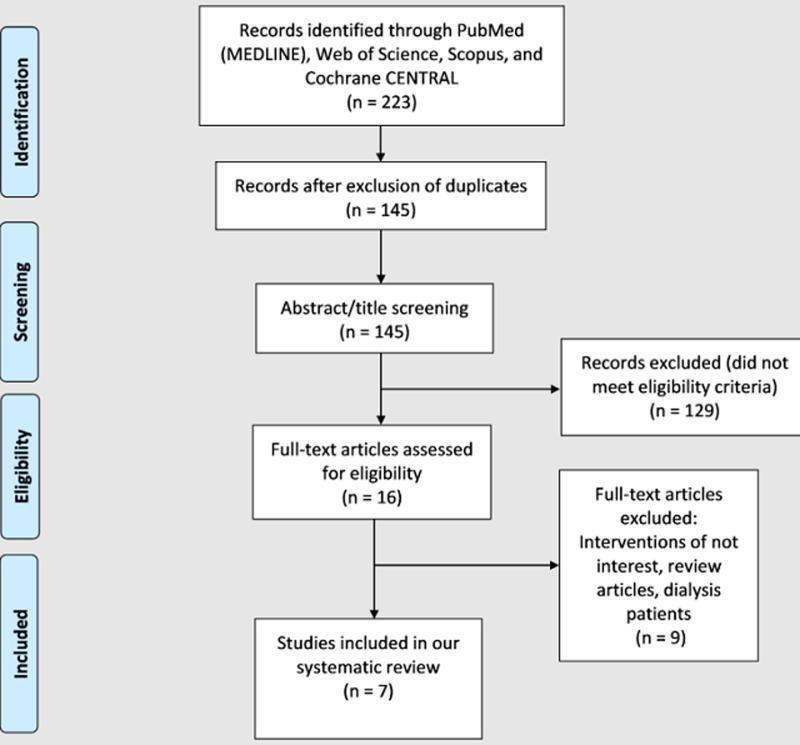
PRISMA flow diagram, which summarizes the literature search, and the number of the obtained records

**Literature search results:** our comprehensive search in databases yielded 223 records; omitting duplicates resulted in 145 unique studies for screening. After screening full texts, seven unique studies were finally included in our systematic review [27]; six entered our NMA [28-33]. We presented the PRISMA flow chart in Figure 1.

**Figure 2:**
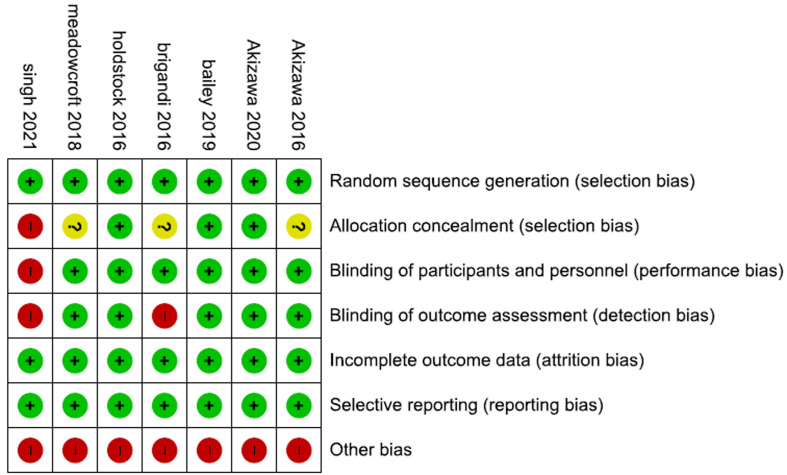
risk of bias graph and summary of the included studies

**Summary and baseline characteristics:** seven multi-center RCTs with a total sample size of 759 comprised the evidence synthesized by this systematic review and NMA. The eligible trials provided data about safety and efficacy outcomes associated with each dose of daprodustat, rhEPO, and placebo. Our assessment of the methodological quality of the included trials showed an overall medium to a high quality of all studies. All eligible trials had a low bias risk regarding the generation of the random sequence, selective, and incomplete reporting. Other bias was high in all studies due to a common funding source (GlaxoSmithKline-GSK-) with a probable conflict of interest (Figure 2).

### Efficacy outcomes

**Figure 3:**
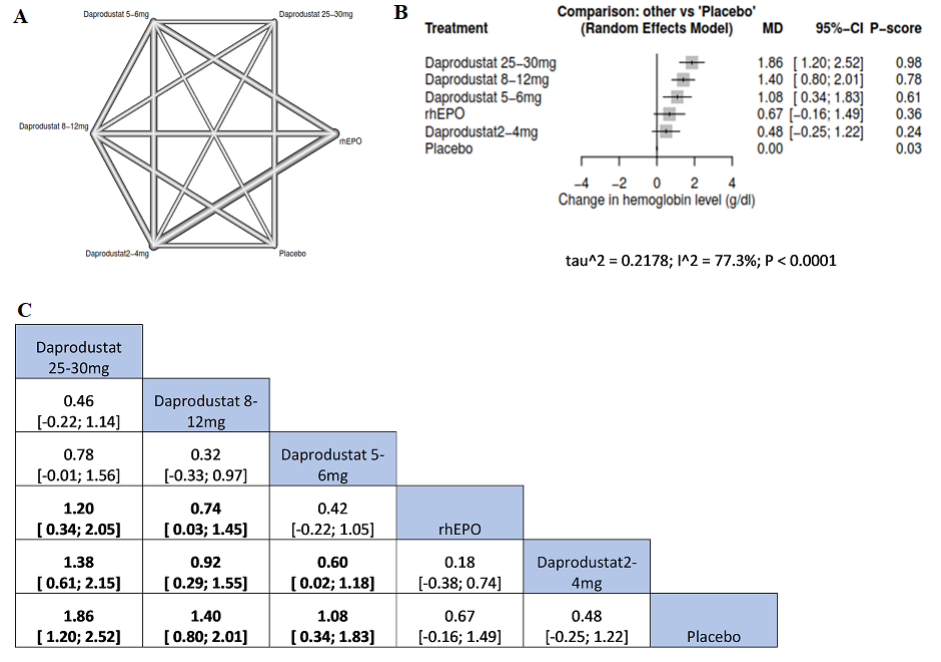
network meta-analysis results of serum HB: (A) network graph showing direct evidence between the assessed interventions; (B) a forest plot was generated by comparing all interventions with “placebo”; P-score was used for ranking; (C) the league table represents the network meta-analysis estimates for all interventions comparisons; the results are the mean difference (MD) with 95% CI, bold items are statistically significant

**Serum hemoglobin (HB):** our results showed that the higher the dose of daprodustat, the significance of the change in serum HB from the baseline. Compared to the placebo, daprodustat 25-30 mg was associated with the highest significant increment in serum HB (MD=1.86, 95%CI= [1.20; 2.52]), followed by daprodustat 8-12 mg (MD= 1.40, 95%CI= [0.80; 2.01]) and daprodustat 5-6mg (MD=1.08, 95%CI= [0.34; 1.83]). Moreover, daprodustat 25-30 mg was associated with more significant rise in serum HB than rhEPO (MD=1.20, 95%CI= [0.34; 2.05]) and daprodustat 2-4mg (MD=1.38, 95%CI= [0.61; 2.15]); daprodustat 8-12mg was reportedly associated with increasing HB more significant than rhEPO (MD= 0.74, 95%CI= [0.03; 1.45]) and daprodustat 2-4 mg (MD=0.92, 95%CI= [0.29; 1.55]); daprodustat 5-6 mg was reportedly associated with more significant increment of HB than daprodustat 2-4 mg (MD=0.60, 95%CI= [0.02; 1.18]) (Figure 3).

**Figure 4:**
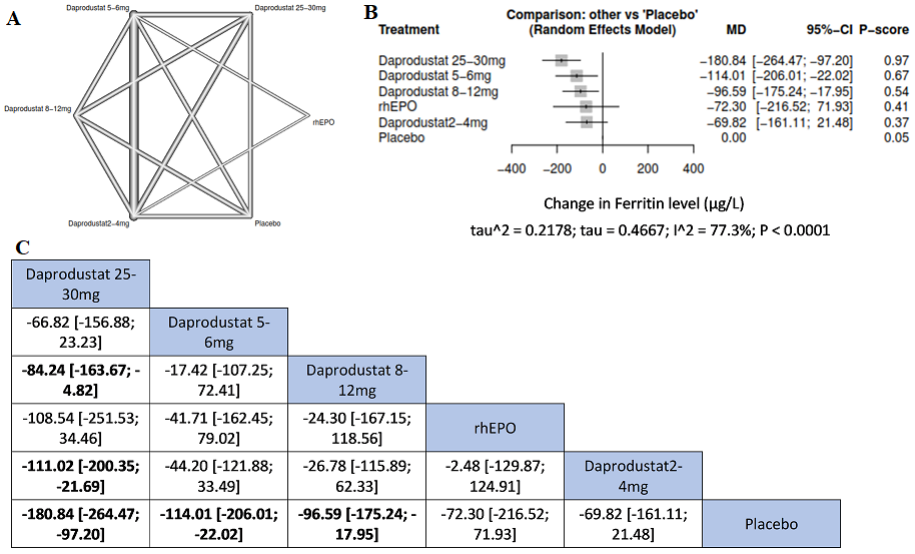
network meta-analysis results of rate of serum ferritin: (A) network graph showing direct evidence between the assessed interventions; (B) a forest plot was generated by comparing all interventions with “placebo”; P-score was used for ranking; (C) the league table represents the network meta-analysis estimates for all interventions comparisons; the results are the mean difference (MD) with 95% CI, bold items are statistically significant

**Serum ferritin:** compared to placebo, daprodustat 25-30 mg was associated with the most significant reduction in serum ferritin (MD= -180.84, 95%CI= [-264.47; -97.20]) followed by daprodustat 5-6 mg (MD= -114.01, 95%CI= [-206.01; -22.02]) and daprodustat 8-12 mg (MD= -96.59, 95%CI= [-175.24; -17.95]). Additionally, daprodustat 25-30 mg was reportedly associated with more significant reduction in serum ferritin than daprodustat 8-12 mg (MD= -84.24, 95%CI= [-163.67; -4.82]) and daprodustat 2-4 mg (MD= -111.02, 95%CI= [-200.35; -21.69]) (Figure 4).

**Figure 5:**
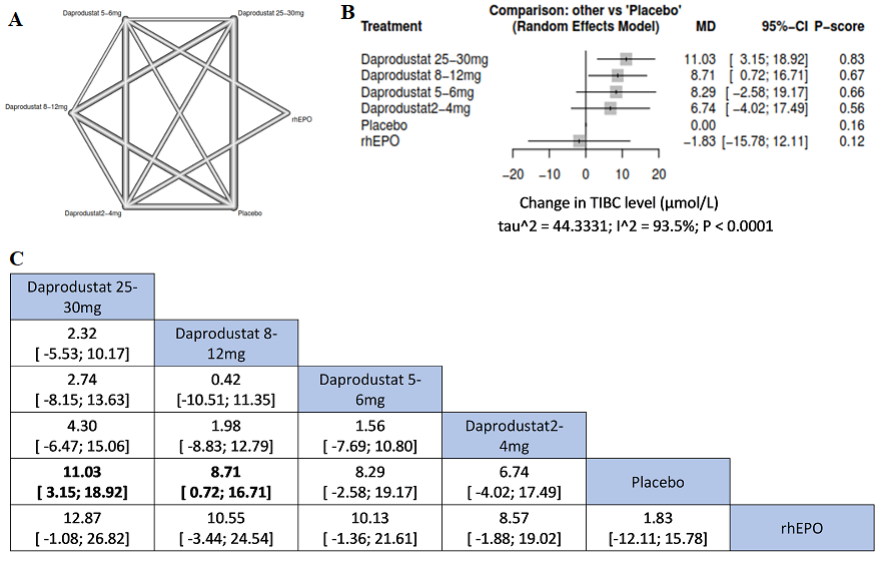
network meta-analysis results of total iron-binding capacity (TIBC): (A) network graph showing direct evidence between the assessed interventions; (B) a forest plot was generated by comparing all interventions with “placebo”; P-score was used for ranking; (C) the league table represents the network meta-analysis estimates for all interventions comparisons; the results are the mean difference (MD) with 95% CI, bold items are statistically significant

**Serum total iron-binding capacity (TIBC):** daprodustat 25-30 mg was associated with more significant increment of serum TIBC than daprodustat 8-12 mg when compared to placebo ((MD=11.03, 95%CI= [3.15; 18.92]), (MD= 8.71, 95%CI= [0.72; 16.71]), respectively) (Figure 5).

**Figure 6:**
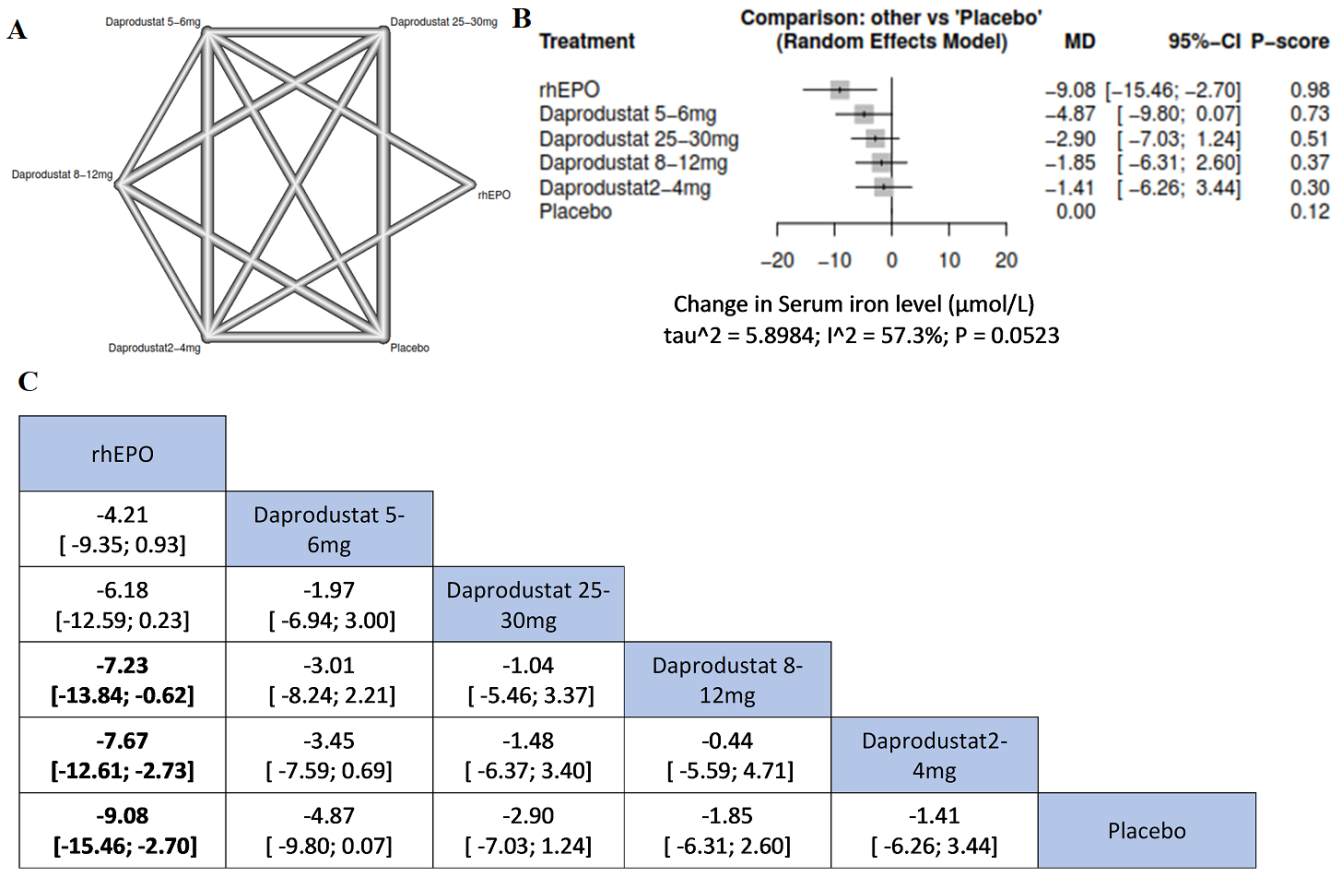
network meta-analysis results of serum iron: (A) network graph showing direct evidence between the assessed interventions; (B) a forest plot was generated by comparing all interventions with “placebo”; P-score was used for ranking; (C) the league table represents the network meta-analysis estimates for all interventions comparisons; the results are the mean difference (MD) with 95% CI, bold items are statistically significant

**Serum iron:** we found that rhEPO administration was associated with the most significant change in serum iron from the baseline; more significant than daprodustat 8-12 mg (MD=-7.23, 95%CI= [-13.84; -0.62]), daprodustat 2-4 mg (MD=-7.67, 95%CI= [-12.61; -2.73]), and placebo (MD= -9.08, 95%CI= [-15.46; -2.70]) (Figure 6).

**Safety outcomes:** Brigandi *et al*. reported that 48% (15 out of 31) of dialysis-dependent patients who intake daprodustat were associated with adverse events (AEs), most commonly hypotension. However, no significant difference was found between daprodustat groups and placebo. Adverse events resulted in the early termination of three patients without any death [29].

Akizawa *et al*. reported that about 30% (32 out of 97) of the enrolled population had AEs, most commonly was nasopharyngitis (total of five patients in different arms of daprodustat) [28]. A study by Meadowcroft *et al*. showed that the incidence of any AEs was proportionate between the control and daprodustat groups. Additionally, about 18% of patients administered daprodustat had serious AEs, most commonly myocardial infarction (MI) and cardiac arrest (each 3 out of 177 patients) [31]. The ASCEND-D study by Singh *et al*. declared that the incidence of a major cardiovascular event (MACE) was about 25.2% in daprodustat compared to 26.7% in ESAs groups (HR= 0.93, 95%CI= [0.81, 1.07]). In addition, they declared that death occurrence was comparable between the two groups [27].

## Discussion

We aimed to conduct a network meta-analysis (NMA) to investigate the direct and indirect effects of different doses of daprodustat, rhEPO, and placebo in dialysis-dependent patients. Our research revealed that daprodustat, at dosages of 25-30 mg, significantly increased serum hemoglobin (HB) and total iron-binding capacity (TIBC), while notably decreasing serum ferritin levels in patients on dialysis with anemia. This finding contrasts with previous reports, such as the meta-analysis by Zhong *et al*. which did not report significant differences. Furthermore, our study highlighted that recombinant human erythropoietin (rhEPO) was linked to the most considerable change in serum iron levels from baseline. The significance of these findings lies in daprodustat's efficacy in improving key hematological parameters in this patient group, challenging earlier findings and suggesting daprodustat as a potent agent for managing anemia in dialysis patients. This contributes valuable insights into the therapeutic landscape for anemia management in dialysis patients, advocating for daprodustat's role in effectively modifying serum HB, TIBC, and ferritin levels, thereby offering a promising alternative to traditional treatments [21]. Our results were consistent with what was reported by the meta-analysis of Zheng *et al*. regarding the daprodustat associated with a significant change in serum TIBC and ferritin. However, they reported a non-significant change in serum HB associated with daprodustat administration, an opposite finding to what our result provided [22].

Literature review revealed many drugs that inhibit HIF-PH, including desidustat, molidustat, vadadustat, enarodustat, roxadustat, and daprodustat; Japan had approved the usage of five HIF-PH inhibitors in the clinical practice [34]. Despite being categorized in the same functional class, all drugs differ in the half-life, the chemical structure, the associated side effects, and the domains of prolyl hydroxylase on which they work [35].

Daprodustat is an orally administered small active molecule that suppresses the first three domains of the enzyme HIF-PH yields an accumulation of HIF-α that dimerizes with HIF-β [35]. The dimer enters the nucleus and activates transcription factors and genes that initiate erythropoiesis and the synthesis of proteins involved in iron absorption, mobilization, and stor [36,37]. From the three discovered isoforms of HIF-α, Iron metabolism was found straightly affected by HIF-1α and HIF-2α [38]; HIF-2α was reportedly associated with a more direct effect on liver metabolism of iron than HIF-1α [39]. HIF-2α was found to upregulate genes of proteins and enzymes that promote intestinal absorption for iron, including divalent transporters and duodenal cytochrome-b. The genes that drive transferrin synthesis was found to be upregulated by HIF-1α, aiding the transport of iron to the tissue [19,39].

The literature showed that HIF-1 was associated with upregulation of vascular-endothelial growth-factor (VEGF) [28]. This protein played a major role in angiogenesis and was reportedly associated with an increased risk of tumor growth and proliferative retinopathy [40,41]. However, many trials had investigated the daprodustat-associated proliferative retinopathy and found no significant change in fundus over the weeks of follow-up [29,30,42]. In addition, daprodustat was reportedly associated with fewer cardiac morbidities than rhEPO [43,44]. This might be due to the significant reduction, nearly physiological, of serum EPO associated with daprodustat administration compared to rhEPO, up to 15 folds less [30,45].

Our study represents the pioneering network meta-analysis (NMA) to evaluate both direct and indirect impacts of various doses of daprodustat, recombinant human erythropoietin (rhEPO), and placebo on anemia in dialysis-dependent chronic kidney disease (CKD) patients. Uniquely, it also marks the first instance of pooling data specifically from 759 dialysis-dependent patients, scrutinizing the effects of different daprodustat doses. Our findings reveal a significant, dose-dependent correlation between daprodustat administration and alterations in serum hemoglobin (HB) and total iron-binding capacity (TIBC), underscoring the therapeutic potential of daprodustat in this context.

Despite these contributions, our research faced limitations, notably in the aggregation of data concerning safety outcomes and certain efficacy metrics like hepcidin and transferrin saturation (TSAT). This was due to the manner in which the included trials reported data, which did not categorize outcomes according to distinct daprodustat dosages. This gap highlights the need for future studies to standardize outcome reporting to facilitate more comprehensive meta-analyses, ensuring a clearer understanding of daprodustat's role in managing anemia among dialysis-dependent CKD patients.

Moreover, our analysis was constrained by the duration of follow-up in four of the included studies, which ranged from four to eight weeks. This limited timeframe hindered our ability to gather data on long-term adverse events, potentially affecting the comprehensiveness of our safety outcomes evaluation. The relatively short follow-up periods underscore the necessity for longer-term studies to assess the enduring safety profile of daprodustat and its effects on anemia in dialysis-dependent CKD patients [28-30,32]. Furthermore, a noteworthy limitation stems from the funding source of the seven included trials, all of which were financed by GlaxoSmithKline (GSK). This financial backing introduces a considerable potential for conflict of interest, raising concerns about the possibility of high bias in the domain of other biases. Such a scenario emphasizes the critical need for transparency and the inclusion of independent studies in future research to mitigate bias and ensure the reliability of findings in the exploration of daprodustat's efficacy and safety in this patient population.

## Conclusion

Our systematic review and network meta-analysis (NMA) mark a significant step forward in evaluating the efficacy of daprodustat across various doses, alongside recombinant human erythropoietin (rhEPO) and placebo, in treating anemia among dialysis-dependent chronic kidney disease (CKD) patients. This research is the first to aggregate and analyze data from 759 such patients, revealing a dose-dependent positive impact of daprodustat on serum hemoglobin (HB) and total iron-binding capacity (TIBC), alongside a reduction in serum ferritin levels. These findings not only challenge previous reports but also position daprodustat as a potentially superior option for managing dialysis-related anemia. Despite the promising results, our study acknowledges limitations, including the short duration of follow-up in some trials and the potential conflict of interest due to industry funding. These factors underline the need for further, independent long-term studies to fully understand daprodustat's safety and efficacy profile.

### What is known about this topic

- *The most crucial cause of CKD-induced anemia is decreased serum erythropoietin (EPO), a hormone that was secreted normally by renal interstitial fibroblast;*
- *The conventional lines of treatments were EPO, EPO stimulating agents (ESA)s, and iron*.

### What this study adds

- *Our study found that daprodustat 25-30 mg was associated with the most significant increment in serum HB and TIBC and the most significant reduction in serum ferritin;*
- *Our study showed that daprodustat was significantly associated with raising serum HB and TIBC and decreasing serum ferritin in patients on dialysis with anemia*.
